# Rotation in Office

**Published:** 1838

**Authors:** 


### Arrt. IX.—Rotation in Office.

The *Boston Medical and Surgical Journal*, gives the following amusing account of the appointment of a physician in chief, to her youthful majesty Queen Victoria. Dr. Clark, the favorite, has commended himself to the profession, by his elaborate work on Pulmonary Consumption. His promotion will give general satisfastion to us republicans of America. It appears that he has been made a Baronet, by the young Queen. D.

*Royal Physicians.*—The Queen of England ascended the throne in June, and one of her first measures was to organize the royal househlod. Lists of persons in each department were prepared, and amongst others, the medical list was submitted to her, headed with *Sir Henry Halford*, *Sir Matthew Tierney*, and some other pillars of that clique of medical exclusives, called the College of Physicians. "*Sir J. Clark* is my physcian," quietly remarked the queen. "Certainly," said a noble official, "his name shall be added to the catalogue." It was then written at the bottom of the list, and the whole again read to her. "There is a mistake," said the queen, "*my physician must come first*, and afterwards you may put on what names you please." So it was done, and Sir J. Clark is the royal physician. But when the official announcement of the medical honor reached the college, they were in a dilemma—for it appeared that "*my physician*" did not belong to that ilk. Mortified and provoked as they were, beyond endurance, to be thwarted in the commencement of a new reign, in monopolizing all the honor and all the fees, as they and their knighted predecessors had done for ages, a show of acquiescence was made, by sending Sir J. Clark a diploma, forthwith. But the medical baronet haughtily refused their parchment honors. It was too late in the day. He had risen to distinction by his own personal industry and excellent character, unaided, uncared for—and, if asked, would probably have said, unknown to the illustrious members of the Royal College. Sir Henry Halford, the president, who has been fed from the royal crib of three kings

(George III. George IV. and William IV.), with gilded oats, is now the *second* physician of the queen.

